# Acknowledgment of Reviewers

**DOI:** 10.2188/jea.JE20120189

**Published:** 2012-11-05

The following scientsts assisted the journal by reviewing manuscripts during the period October 1, 2011 to September 30, 2012. We gratefully acknowledge their critical evaluation and assistance in selecting articles for publication.

**Table:** 

Adachi, Hisashi	Kurume University
Aida, Jun	Tohoku University
Akiba, Suminori	Kagoshima Univeristy
Arao, Takashi	Waseda University
Arima, Hisatomi	The George Institute
Arisawa, Kokichi	The University of Tokushima
Asao, Keiko	University of Michigan Health System
Babazono, Akira	Kyushu University
Davaalkham, Dambadarjaa	Health Sciences University of Mongolia
Doi, Yasufumi	Kyushu University
Doi, Yuriko	National Institute of Public Health
Eguchi, Hidetaka	Saitama Medical University
Fellman, Johan	Folkhälsan Institute of Genetic
Foo, Leng	Universiti Sains Malaysia
Fujita, Yuki	Kinki University
Fukuda, Yoshiharu	Yamaguchi Unversity
Furuna, Taketo	Sapporo Medical University
Goto, Aya	Fukushima Medical University
Hamajima, Nobuyuki	Nagoya University
Hara, Kazuo	The University of Tokyo Hospital
Hara, Megumi	Saga University
Hashimoto, Hideki	The University of Tokyo
Hashizume, Masahiro	Institute of Tropical Medicine, Nagasaki University
Hayashi, Kunihiko	Gunma University
Hayashida, Kenshi	University of Occupational and Environmental Health
Hayashino, Yasuaki	Kyoto University
Higashi, Takahiro	The University of Tokyo
Hirose, Kaoru	Aichi Prefectural Institute of Public Health
Hishida, Asahi	Nagoya University
Hodgson, M. Elizabeth	University of North Carolina
Honda, Sumihisa	Nagasaki University
Honda, Yasushi	University of Tsukuba
Honjo, Kaori	Osaka University Global Collaboration Center
Honjo, Satoshi	Fukuoka East Medical Centre
Hoshuyama, Tsutomu	Ushibuka-Shimin Hospital
Hosono, Satoyo	Aichi Cancer Center Research Institute
Hozawa, Atsushi	Tohoku Medical Megabank Organization
Ichikawa, Masao	University of Tsukuba
Ikeda, Ai	National Cancer Center
Imaeda, Nahomi	Nagoya Women’s University
Imamura, Fumiaki	Harvard School of Public Health
Ishihara, Junko	Sagami Women’s University
Ishikawa, Hirofumi	Okayama University
Ishizaki, Tatsuro	Tokyo Metropolitan Institute of Gerontology
Ito, Yuri	Osaka Medical Center for Cancer and Cardiovascular Disease
Kadotani, Hiroshi	Kyoto University
Kadowaki, Takashi	Shiga University of Medical Science
Kai, Ichiro	The University of Tokyo
Kakehashi, Masayuki	Hiroshima University
Kaku, Mitsuo	Tohoku University
Kanda, Hideyuki	Fukushima Medical University
Kaneko, Yoshihiro	Akita University
Katanoda, Kota	National Cancer Center
Kawakami, Norito	The University of Tokyo
Kawamura, Takashi	Kyoto University
Kayaba, Kazunori	Saitama Prefectural University
Kikuchi, Shogo	Aichi Medciacl University School of Medicine
Kobashi, Gen	National Institute of Radiological Sciences
Kojima, Masayo	Nagoya City University
Kokubo, Yoshihiro	National Cardiovascular Center
Kondo, Naoki	The University of Tokyo
Kondo, Takaaki	Nagoya University
Kotani, Kazuhiko	Jichi Medical University
Kubo, Tatsuhiko	University of Occupational and Environmental Health
Kuriyama, Nagato	Kyoto Prefectural University of Medicine
Kuriyama, Shinichi	Tohoku University
Lee, Kyoung-Mu	Korea National University
Li, Yan	Shanghai Institute of Hypertension
Liao, Chung-Min	National Taiwan University
Lim, Wei-Yen	National University of Singarpore
Lin, Yingsong	Aichi Medical Uniersity
Ma, Enbo	University of Tsukuba
Matsuda, Shinya	University of Occupational and Environmental Health
Matsuda, Tomohiro	National Cancer Center
Matsumura, Yasuhiro	Bunkyo University
Matsuo, Keitaro	Aichi Cancer Center Research Institute
Matsushima, Masato	Jikei University School of Medicine
Matsushita, Yumi	National Center for Global Health and Medicine
Metoki, Hirohito	Tohoku University School of Medicine
Miyachi, Motohiko	National Institute of Health & Nutrition
Miyake, Yoshihiro	Fukuoka University
Miyamatsu, Naomi	Shiga University of Medical Science
Mochizuki, Yoshikatsu	Asahikawa Medical University
Mori, Mitsuru	Sapporo Medical University
Morikawa, Yuko	Kanazawa Medical University
Morino, Yoshihiro	Iwate Medical University
Morita, Akemi	National Institute of Health and Nutrition
Morita, Manabu	Okayama University
Murakami, Yoshitaka	Shiga University of Medical Science
Murata, Chiyoe	Hamamatsu University School of Medicine
Murayama, Hiroshi	Tokyo Metropolitan Institute of Gerontology
Nagai, Masaki	Saitama Medical University
Nagano, Jun	Kyushu University
Nagata, Chisato	Gifu University
Nakamura, Kazutoshi	Niigata University
Nakamura, Koshi	Kanazawa Medical University
Nakamura, Mieko	Hamamatsu University
Nakamura, Yasuyuki	Kyoto Women’s University
Nakamura, Yosikazu	Jichi Medical University
Nakata, Yoshio	University of Tsukuba
Nakaya, Naoki	Kamakura Women’s University
Nakayama, Takeo	Kyoto University
Nishi, Daisuke	National Disaster Medical Center
Nishino, Yoshikazu	Miyagi Cancer Center Research Institute
Nishiura, Chihiro	Tokyo Gas Co., Ltd.
Nishiwaki, Yuji	Toho University
Nishiyama, Takeshi	Nagoya City University
Nojima, Masanori	Sapporo Medical University
Obara, Taku	Tohoku University
Ohira, Tetsuya	Osaka University
Ohkado, Akihiro	Japan Anti-Tuberculosis Association
Ohsawa, Masaki	Iwate Medical University
Ohshige, Kenji	Yokohama National University
Ojima, Toshiyuki	Hamamatsu University School of Medicine
Okada, Masafumi	University of Tsukuba
Okamoto, Etsuji	National Institute of Public Health
Okamoto, Kazushi	Aichi Prefectural University
Okuda, Nagako	Shiga University of Mecdical Science
Okumura, Junko	Institute of Tropical Medicine, Nagasaki University
Ooki, Syuichi	Ishikawa Prefectural Nursing University
Osaki, Yoneatsu	Tottori University
Oshitani, Hitoshi	Tohoku University
Otsuka, Rei	National Center for Geriatrics and Gerontology
Ozasa, Kotaro	Radiation Effects Research Foundation, Japan
Oze, Isao	Aichi Cancer Center Research Institute
Pham, Truong-Minh	Radiation Effects Research Foundation, Japan
Saijo, Yasuaki	Asahikawa Medical College
Sairenchi, Toshimi	Dokkyo Medical University
Saito, Isao	Ehime University
Sakamoto, Naoko	National Research Institute for Child Health & Development
Sakurai, Masaru	Kanazawa Medical University
Sasaki, Satoshi	The University of Tokyo
Sato, Shinichi	Chiba Prefectural Institute of Public Health
Sawada, Norie	National Cancer Center
Sawada, SS	Tokyo Gas Co., Ltd.
Schernhammer, Eva	Harvard Medical School
Sekikawa, Akira	University of Pittsburgh
Setoguchi, Soko	Duke Clinical Research Institute
Shima, Masayuki	Hyogo College of Medicine
Shimazu, Taichi	National Cancer Center
Shimokata, Hiroshi	National Institute for Geriatrics and Gerontology
Shirai, Kokoro	Harvard Schoool of Public Health
Sone, Hirohito	University of Tsukuba
Sonobe, Tomoyoshi	Japanese Red Cross Medical Center
Suganuma, Narufumi	Kochi Medical School
Sugimura, Haruhiko	Hamamatsu University School of Medicine
Sugisawa, Hidehiro	J. F. Orberlin University
Sumi, Ayako	Sapporo Medical University
Suzuki, Kohta	University of Yamanashi
Tabara, Yasuharu	Kyoto University
Takahashi, Masaya	Japan NIOSH
Takao, Soshi	Okayama University
Takebayashi, Toru	Keio University
Takeshita, Tatsuya	Wakayama Medical University
Takezaki, Toshiro	Kagoshima University
Tamaki, Junko	Kinki University
Tamakoshi, Akiko	Hokkaido University
Tamakoshi, Koji	Nagoya University
Tamashiro, Hiko	Hokkaido University
Tanabe, Naohito	Niigata University
Tanaka, Chiaki	J. F. Oberlin University
Tanaka, Hideo	Aichi Cancer Center Research Institute
Tanaka, Junko	Hiroshima University
Tanaka, Keitaro	Saga University
Tanaka, Shiro	Kyoto University Hospital
Tanihara, Shinnichi	Fukuoka University
Tanimura, Susumu	Hyogo College of Medicine
Teramoto, Shinji	Hitachinaka Medical Education and Research Center, The University of Tsukuba
Teramukai, Satoshi	Kyoto University Hospital
Toyoshima, Hideaki	Anjo Kosei Hospital
Tsugane, Shoichiro	National Cancer Center
Tsukuma, Hideaki	Osaka Medical Center for Cancer and Cardiovascular Disease
Tsutsumi, Akizumi	University of Occupational and Environmental Health
Ueda, Kayo	National Institute for Environmental Studies
Ueji, Masaru	Ibaraki University
Umesawa, Mitsumasa	Center for Medical Sciences
Wakai, Kenji	Nagoya University
Washio, Masakazu	St. Mary’s College
Watanabe, Makoto	National Cardiovascular Center
Watanabe, Shuichiro	J. F. Oberlin University
Yamaji, Taiki	National Cancer Center
Yamamoto, Seiichiro	National Cancer Center
Yamauchi, Hideko	St. Luke’s International Hospital
Yamauchi, Takashi	National Center of Neurology and Psychiatry
Yanagita, Masahiko	Doshisha University
Yanai, Hideki	Fukujuji Hospital
Yatsuya, Hiroshi	Fujita Health University
Yokoyama, Tetsuji	National Institute of Public Health
Yonemoto, Naohiro	National Center of Neurology and Psychiatry
Yoshiike, Nobuo	Aomori University of Health and Welfaire
Yoshimi, Itsuro	Tokyo Metropolitan Government
Yoshimura, Noriko	The University of Tokyo
Yoshinaga, Masao	National Hospital Organization Kagoshima Medical Center

